# Acute stress triggers sex-dependent rapid alterations in the human small intestine microbiota composition

**DOI:** 10.3389/fmicb.2024.1441126

**Published:** 2025-01-15

**Authors:** Bruno K. Rodiño-Janeiro, Olfat Khannous-Lleiffe, Marc Pigrau, Jesse R. Willis, Eloísa Salvo-Romero, Adoración Nieto, Elba Expósito, Marina Fortea, Cristina Pardo-Camacho, Mercé Albert-Bayo, Ana María González-Castro, Danila Guagnozzi, Cristina Martínez, Beatriz Lobo, María Vicario, Javier Santos, Toni Gabaldón, Carmen Alonso-Cotoner

**Affiliations:** ^1^Laboratory of Neuro-Immuno-Gastroenterology, Digestive System Research Unit, Vall d'Hebron Institut de Recerca (VHIR), Vall d'Hebron Hospital Universitari, Vall d'Hebron Barcelona Hospital Campus, Barcelona, Spain; ^2^Department of Gastroenterology, Vall d'Hebron Hospital Universitari, Vall d'Hebron Barcelona Hospital Campus, Barcelona, Spain; ^3^Barcelona Supercomputing Centre (BSC-CNS), Barcelona, Spain; ^4^Institute for Research in Biomedicine (IRB Barcelona), The Barcelona Institute of Science and Technology, Barcelona, Spain; ^5^Facultat de Medicina, Universitat Autònoma de Barcelona, Bellaterra, Spain; ^6^Laboratory of Translational Mucosal Immunology, Vall d'Hebron Institut de Recerca (VHIR), Vall d'Hebron Hospital Universitari, Vall d'Hebron Barcelona Hospital Campus, Barcelona, Spain; ^7^Centro de Investigación Biomédica en Red de Enfermedades Hepáticas y Digestivas (CIBEREHD), Instituto de Salud Carlos III, Madrid, Spain; ^8^Renal Physiopathology Group, Vall d'Hebron Institut de Recerca (VHIR), Vall d'Hebron Hospital Universitari, Vall d'Hebron Barcelona Hospital Campus, Barcelona, Spain; ^9^Catalan Institution for Research and Advanced Studies (ICREA), Barcelona, Spain; ^10^Centro de Investigación Biomédica En Red de Enfermedades Infecciosas (CIBERINFEC), Instituto de Salud Carlos III, Madrid, Spain

**Keywords:** stress, functional dyspepsia, disorders of gut-brain interaction, irritable bowel syndrome, small intestine microbiota

## Abstract

**Background/aims:**

Digestive disorders of gut-brain interaction (DGBI) are very common, predominant in females, and usually associated with intestinal barrier dysfunction, dysbiosis, and stress. We previously found that females have increased susceptibility to intestinal barrier dysfunction in response to acute stress. However, whether this is associated with changes in the small bowel microbiota remains unknown. We have evaluated changes in the small intestinal microbiota in response to acute stress to better understand stress-induced intestinal barrier dysfunction.

**Methods:**

Jejunal biopsies were obtained at baseline and 90 min after cold pain or sham stress. Autonomic (blood pressure and heart rate), hormonal (plasma cortisol and adrenocorticotropic hormone) and psychological (Subjective Stress Rating Scale) responses to cold pain and sham stress were monitored. Microbial DNA from the biopsies was analyzed using a 16S metabarcoding approach before and after cold pain stress and sham stress. Differences in diversity and relative abundance of microbial taxa were examined.

**Results:**

Cold pain stress was associated with a significant decrease in alpha diversity (*P* = 0.015), which was more pronounced in females, along with significant sex differences in the abundance of specific taxa and the overall microbiota composition. Microbiota alterations significantly correlated with changes in psychological responses, hormones, and gene expression in the intestinal mucosal. Cold pain stress was also associated with activation of autonomic, hormonal and psychological response, with no differences between sexes.

**Conclusions:**

Acute stress elicits rapid alterations in bacterial composition in the jejunum of healthy subjects and these changes are more pronounced in females. Our results may contribute to the understanding of female predominance in DGBI.

## 1 Introduction

Irritable bowel syndrome (IBS) and functional dyspepsia (FD) are major disorders of gut-brain interaction (DGBI) that predominantly affect females (Sperber et al., 2021). The main symptoms have been linked to intestinal barrier (IB) dysfunction (Santos and Rescigno, 2024), neuroimmune activation (Vanuytsel et al., 2023), dysbiosis and small bowel bacterial overgrowth (SIBO) (Barlow et al., 2021; Gurusamy et al., 2021; Saffouri et al., 2019), stress, and psychological factors. These patients often suffer from difficult-to-treat comorbid chronic pain and disability disorders such as fibromyalgia, chronic fatigue and depression/anxiety (Ohlsson, 2022; Van Oudenhove and Aziz, 2013) that highly impact healthcare costs and the patient's quality of life (Agarwal and Spiegel, 2011; Nyrop et al., 2007).

The human gut is colonized by diverse microbial communities, collectively known as the gut microbiota (Lynch and Pedersen, 2016). This microbiota plays a key role in promoting intestinal health, and when altered, is associated with the development of gastrointestinal (GI) symptoms in DGBI. Such a relationship is supported by good long-term clinical outcomes of fecal microbial transplantation in IBS patients (El-Salhy et al., 2022). However, most previous studies in DGBI relied on fecal or mucosal samples from the large bowel, and the composition and the role of small bowel microbiota remains largely unknown despite recent advances in both IBS and FD (Kastl et al., 2020; Vasapolli et al., 2019; Shanahan et al., 2022; Rodiño-Janeiro et al., 2018). Nevertheless, it is noteworthy that small intestinal microbiota dysbiosis, rather than SIBO, may be responsible for the main GI symptoms in DGBI (Saffouri et al., 2019).

The gut barrier could be a possible explanation for the link between stress, gut dysbiosis, and the female sex. On one side, previous research has shown that both chronic psychosocial stress and female sex lead to a significant IB dysfunction in response to cold pain stress (CPS) in healthy subjects (Alonso et al., 2008, 2012; Vanuytsel et al., 2014). On the other, it has been shown that gut dysbiosis is involved in the development of barrier dysfunction in the post-infectious (PI) variants of IBS and FD (PI-IBS & PI-FD) (Mearin et al., 2005; Marshall et al., 2010) where female sex and comorbid psychological conditions are among the most significant risk factors. In fact, multiple mechanisms related to intestinal dysbiosis can contribute to intestinal barrier dysfunction in DGBI. These include the production of dozens of neurotransmitters (Strandwitz, 2018) and endocrine mediators (Furness et al., 2013) and a myriad of metabolites, named collectively as the gut-brain connectome (Sasso et al., 2023) that are known to regulate the vast immune system associated to the gut mucosa (Pabst et al., 2008), the enteric and autonomous system (Gershon and Margolis, 2021) and brain physiology (Sasso et al., 2023), all of which participate in the modulation of IB. Some of the relevant microbiota-derived molecules are the short chain fatty acids, such as butyrate, indoles, tryptophan, aryl hydrocarbon receptor ligands, proteases, lipopolysaccharide from the bacterial wall or deconjugated bile acids (Trzeciak and Herbet, 2021; Gasaly et al., 2021). Butyrate, for instance, a major energy resource of intestinal epithelial cells, has been shown to regulate the assembly of tight junctions through the activation of AMP-activated protein kinase (Peng et al., 2009) or to inhibit NLRP3 inflammasome activation and autophagy, protecting the intestinal barrier from LPS disruption (Feng et al., 2018). Moreover, probiotics help to strengthen the intestinal barrier through an increase in the expression of occluding (Qin et al., 2005) and ZO-2, and prevent apoptosis of intestinal epithelial cells in a model of dextran sulfate sodium colitis which leads to a stabilization of the epithelial barrier (Mennigen et al., 2009). More recently, a mouse model of water avoidance stress (WAS), has shown that stress inhibited the NLPR6 inflammasome, which is necessary for the homeostasis of the gut microbiome, and that the use of probiotics reversed this effect (Sun et al., 2013).

Stress, both acute and chronic, has also been shown to disrupt the IB and local immune response, including mast cell (MC) activation, in DGBI patients (Santos et al., 1998; Wallon et al., 2008; Gareau et al., 2007; Söderholm et al., 2002; Demaude et al., 2006) In both FD (Wauters et al., 2022) and IBS (Hanning et al., 2021), increased epithelial permeability may facilitate excessive transepithelial antigen penetration, uncontrolled immune activation, and intestinal microinflammation. Although the exact mechanisms by which stress impacts gut physiology are poorly understood, intestinal dysbiosis may be one of them.

To fill this important gap, we here set out to assess changes in small intestinal microbiota that may contribute to IB dysfunction in response to acute stress, and evaluate possible differences in these changes between males and females.

## 2 Materials and methods

### 2.1 Participants

Healthy subjects (18–50 years-old) were prospectively recruited by public advertising. Candidates were asked to fill out the Modified Social Readjustment Scale of Holmes-Rahe (to evaluate significant life events in the last year) (Holmes and Rahe, 1967) and the Beck's inventory for depression (to assess depression levels in the last week) (Beck et al., 1961) prior to entering the study, and a complete medical history and physical examination were carried out on all candidates. Inclusion criteria were to be healthy with not known chronic health disorders, age between 18 and 50 years, signature of informed consent, negative pregnancy test on the day of biopsy, and presence of regular menses in the absence of oral contraceptives. Exclusion criteria included history of acute gastroenteritis in the last 2 years, regular smoking, history of abnormal menstrual cycle, pregnancy at the time of the biopsy, usual contraceptive use, and suffering from any chronic health disorders. Once included in the study protocol, subjects were not allowed to take salicylates, non-steroidal anti-inflammatory drugs (NSAIDs), anticholinergic drugs or opioids at least 15 days prior to the biopsy. Antibiotics and probiotics were not allowed in the 2 months prior to inclusion.

The study protocol was approved by the Ethics Committee at Hospital Vall d'Hebron (PR(IR)149/2016) and conducted according to the revised Declaration of Helsinki. All subjects gave their written informed consent.

### 2.2 Jejunal biopsy

Two consecutive mucosal biopsies were obtained from the proximal jejunum using a Watson's capsule: the first at baseline (PRE) and the second, 90 min after completing the 15 min-period of CPS (POST). Tissue samples were immediately split into two similar pieces with a sterile scalpel. One piece was fixed in formalin and embedded in paraffin for routine histology and the other was placed in RNA later (Ambion, Invitrogen), kept at 4°C for 2 h and stored at −80°C until processed for DNA isolation.

### 2.3 Cold pain stress

Acute experimental stress was induced by the cold-water pressor test (Lovallo, 1975). Briefly, participants immersed the non-dominant hand in iced water (4°C) for periods of 45 s followed by 15-s withdrawal intervals, to prevent adaptation to pain, for a total time of 15 min.

### 2.4 Systemic response to cold pain stress

#### 2.4.1 Hand pain perception

The level of hand discomfort/pain was assessed using a visual analog scale from 0 (no discomfort) to 10 (intolerable pain).

#### 2.4.2 Autonomic response

Autonomic response was evaluated by measuring blood pressure and heart rate (HR) with an automated sphygmomanometer (Omron M4-I, Omron Healthcare Europe B.V., Hoofddorp, Netherlands).

#### 2.4.3 Psychological response

The level of acute stress experienced by participants was evaluated by the Subjective Stress Rating Scale (SSRS) (Naliboff et al., 1991).

#### 2.4.4 Hormonal response

Hypothalamic–pituitary–adrenal axis activation was assessed through plasma levels of adrenocorticotropic hormone (ACTH) and cortisol.

### 2.5 Experimental design

Physical examination and assessment of baseline stress and depression levels were performed the week prior to the jejunal biopsy. After an overnight fast, participants were orally intubated at 8:00 h and a first biopsy baseline biopsy (PRE) was collected at 5 cm distal to the Treitz's angle. Thereafter, the capsule was withdrawn and subjects were intubated, again with a second Watson's capsule, submitted to the CPS protocol and 90 min after finishing CPS a second biopsy obtained from the same location was taken (POST).

**Figure 1:**
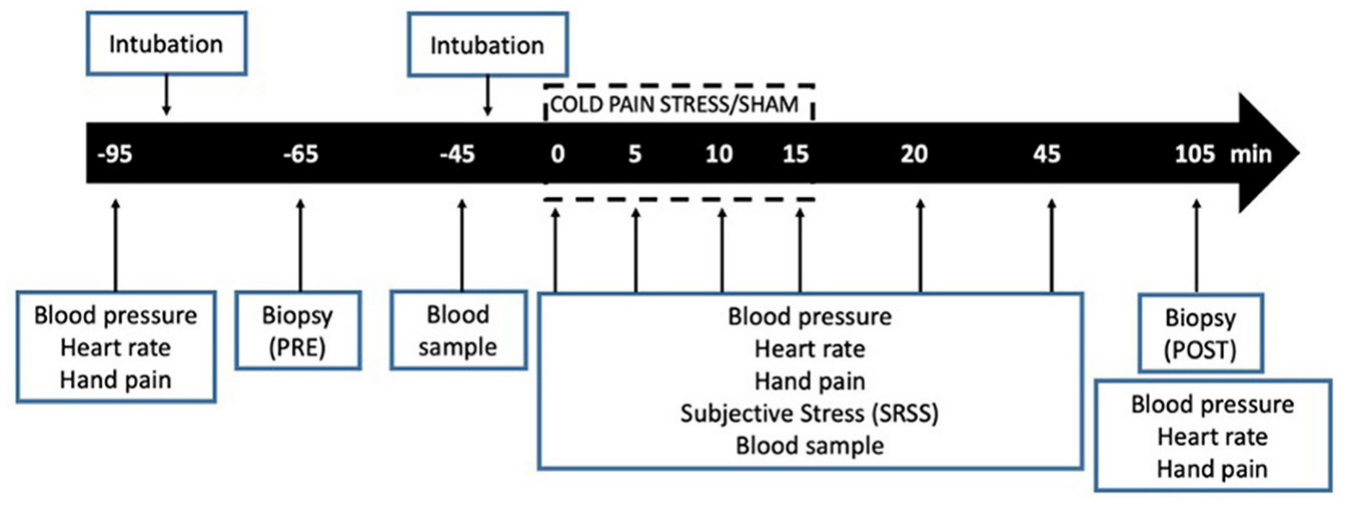
Experimental design.

Blood samples (20 mL) were collected before oral intubation (t−95), after collecting baseline biopsy (t−45), immediately before initiating CPS (t0), 5, and 15 min after stress initiation (t 5, t15) and 30 (t45) and 90 (t105) min after stress cessation. Autonomic and psychological responses were measured before oral intubation (t−95), once intubated (t−65), and after collecting baseline biopsy (t−45), immediately before initiating CPS (t0), 5, 10 15 and 20 min after stress initiation (t5, t10, t15, t20) and 30 (t45) and 90 (t105) min after stress cessation. Hand pain perception was assessed before initiating CPS (t0), every 5 min until finishing CPS (t5, 10 and 15), and 5 (t20) and 30 min (t45) after stress cessation (Figure 1).

To evaluate whether the changes observed could be related to the intubation itself, a sham stress protocol was also performed in a reduced number of participants, using the same procedures, but with no CPS. Intestinal biopsies, blood collection and processing of samples were performed identically in both groups.

### 2.6 DNA extraction, library preparation and sequencing

DNA isolation was performed from the whole biopsy (50–100 mg) using the TRIzol method. In brief, the sample was homogenized in TRIzol using the Lysing Matrix D (MP Biomedicals) in FP120A FastPrep (Thermo electron). The homogenate was passed through a 27G syringe and chloroform was added. The homogenate was centrifuged to divide the aqueous from the organic phase. The organic phase contains the tissular protein and DNA. The DNA was precipitated from the organic phase using 100% ethanol. The precipitated DNA was washed with 0.1 M sodium citrate in 10% ethanol at pH 8.5 three times. A final wash with ethanol 75% was performed and the pellet was dried at room temperature for 15 min. Finally, the DNA was resuspended in 8 mM NaOH, 10mM HEPES and 1mM EDTA. After the isolation, the DNA samples were stored at −80°C.

Samples were amplified using primers for the V3–V4 regions: forward: (5′-TCG TCG GCA GCG TCA GAT GTG TAT AAG AGA CAG CCT ACGGGNGGC WGC AG-3′), reverse: (5′GTC TCG TGG GCT CGG AGA TGT GTA TAA GAG ACA GGA CTACHVGGG TAT CTA ATC C-3′).

The PCR was performed in 10-μl final volume with 0.2 μM primer concentration.

1) 1st PCR: 3 min at 95°C (initial denaturation) followed by 30 cycles: 30 s at 95°C, 30 s at 55°C, and 30 s at 72°C. Final elongation step of 5 min at 72°C. PCR products were purified with AMPure XP beads (Beckman Coulter, Nyon, Switzerland) with a 0.9 × ratio according to the manufacturer's instructions. PCR products were eluted from the magnetic beads with 30 μl Milli-Q water.
2) 2nd PCR: 5 μl of the first PCR purified product were used as the template for a second PCR with Nextera XT v2 adaptor primers in a final volume of 30 μl using the same PCR mix and thermal profile as for the first PCR but with only eight cycles. Twenty-five microliter of the second PCR product were purified with SequalPrep normalization kit (Invitrogen, ThermoFisher Scientific, Waltham, MA, USA), according to the manufacturer's protocol.
3) Sequencing: Libraries were eluted in 20 μl and pooled. Sequencing was performed in an Illumina (MiSeq 2 × 300 bp, v3 chemistry). ZymoBIOMICS Microbial Community DNA Standard (ZymoResearch) was used as a positive control. Samples were sequenced in two batches (named 2018 and 2019), libraries for some samples rendering a low number of reads were sequenced in the two batches, and the sequencing runs were combined into a single file (combined).

### 2.7 Hormonal response

Plasma adrenocorticotropic hormone (ACTH) was determined by sandwich chemiluminescence immunoassay (LaisonXL, DiaSorin S.p.A., Saluggia, Italy) and cortisol concentration, measured by chemiluminescent immunoassay (ADVIA Centaur Cortisol assay, Siemens Healthcare Diagnostics, Munich, Germany). Blood samples were collected in plastic tubes (BD Vacutainer^®^ Plus Plastic K2-EDTA Tubes, Franklin Lakes, NJ, USA), centrifuged and aliquoted for hormonal determinations.

### 2.8 Microbiome analysis

Taxonomy assignment was obtained from sequencing data using the dada2 (v. 1.10.1) (Callahan et al., 2016) pipeline.

Quality was assessed with the plotQualityProfile function. The filterAndTrim function was used to filter out or trim low-quality sequencing reads, as well as to remove the first 10 nucleotides (corresponding to the adaptors). The following parameters were used for each sample group, according to the sequencing run (2018, 2019, and combined):

- filterAndTrim (fnFs.2018, filtFs.2018, fnRs.2018, filtRs.2018, truncLen = c(260,230), maxN = 0, maxEE = c(10,10), truncQ = 2, rm.phix = TRUE, trimLeft = c(10,10), compress = TRUE, multithread = TRUE).
- filterAndTrim (fnFs.2019, filtFs.2019, fnRs.2019, filtRs.2019, truncLen = c(280,240), maxN = 0, maxEE = c(10,10), truncQ = 2, rm.phix = TRUE, trimLeft = c(10,10), compress = TRUE, multithread = TRUE).
- filterAndTrim (fnFs.combined, filtFs.combined, fnRs.combined, filtRs.combination, truncLen = c(270,220), maxN = 0, maxEE = c(10,10), truncQ = 1, rm.phix = TRUE, trimLeft = c(10,10), compress = TRUE, multithread = TRUE).

Then, identical sequencing reads were combined into unique sequences to avoid redundant comparisons (dereplication), sample sequences were inferred (from a pre-calculated matrix of estimated learning error rates) and paired reads were merged to obtain full denoised sequences. From these, chimeric sequences were removed. Taxonomy was assigned to ASVs using the SILVA 16s rRNA database (v. 132) (Quast et al., 2013). Next, a phylogenetic tree representing the taxa found in the sample dataset was reconstructed by using the phangorn (v. 2.5.5) (Schliep, 2011) and Decipher R packages (v 2.10.2) (Wright, 2016). We integrated the information from the ASV table, Taxonomy table, phylogenetic tree and metadata (information relative to the samples such as the sex, stress group according to Holmes-Rahe score, age, etc) to create a phyloseq (v. 1.26.1) (McMurdie and Holmes, 2013) object. As mentioned above, positive controls (mock communities) were sequenced and included in the ASV table to evaluate the accuracy of the pipeline, but were not included in subsequent statistical analyses.

The metadata (Supplementary Table S15) consisted of 88 variables including information about the volunteer such as the sex, age, autonomic, hormonal and psychological (measured by the Subjective Stress Rating Scale, SSRS) response to CPS. We also used the fold change (FC) after CPS of genes related to epithelial barrier (CLDN1, CLDN2, OCL, SLC26A3, TJP1 and TJP3), stress regulation and circadian rhythm (NR3C1, NR1D1, NR1D2, PER1 and PER3), inflammation (SOD1, IL18, NFE2L2, NIFL3) and mast cell activation (protease inactivation (SERPINA1), tryptase: TPSAB1) from a previous expression analysis performed on the same intestinal samples (Rodiño-Janeiro et al., 2017, 2023). We created one variable (SeqGroup) to account for the sequencing run, with the labels 2018_only, 2018_reseq, 2019_only and 2019_reseq (including the term reseq for those samples that were sequenced in the two runs).

We characterized the taxonomic composition by calculating different alpha-diversity (within-sample) and beta-diversity (between samples) metrics. Using the estimate_richness function of the phyloseq package we calculated various alpha diversity metrics, including Observed (number of unique species), Shannon (accounting for both the number of species and their relative abundances) and Simpson (which focuses more on dominant species) indices, and also the Chao1, InvSimpson and Abundance-based Coverage Estimator (ACE) metrics. We also computed an alpha diversity metric that incorporates branch lengths of the phylogenetic tree called Faith's phylogenetic diversity by using the picante package (v.1.8.1) (Kembel et al., 2010). Regarding beta-diversity, we used the Phyloseq and Vegan (v. 2.5-6) packages (Oksanen et al., 2024) to characterize nine distance metrics based on the differences in taxonomic composition of the samples including Jensen-Shannon divergence (JSD), Weighted-Unifrac, Unweighted-Unifrac, VAW-Gunifrac, a0-Gunifrac, a05_Gunifrac, Bray, Jaccard and Canberra. We also computed the Aitchison distance (Gloor et al., 2017) using the cmultRepl and codaSeq.clr functions from the CodaSeq (v. 0.99.6) (Gloor and Reid, 2016) and zCompositions (v.1.3.4) (Palarea-Albaladejo and Martín-Fernández, 2015) packages. Normalization was performed by transforming the data to relative abundances, samples containing fewer than 950 reads were discarded and taxa that appeared in fewer than 5% of the samples were filtered out.

### 2.9 Statistical analysis

Clinical data are expressed as median with first and third quartiles (Q1–Q3), unless otherwise stated. Comparisons were made through parametric (paired and unpaired Student's *t*-test and Pearson's correlation) or non-parametric tests (Mann–Whitney *U*-test, Wilcoxon Signed Ranks test, Fisher's exact test and Spearman's correlation coefficient) as appropriate. Hormonal, autonomic, and psychological changes were compared using a two-way repeated-measures Analysis of Variance (ANOVA) where Sex was considered the between-subjects factor, and changes throughout perfusion time the within-subject factor (Time). Mean imputation was used for random missing values when needed.

We investigated the microbial composition of the samples using a 16S metabarcoding approach. In total, 68 out of the 74 sequenced samples passed all quality filters and were considered for subsequent analysis. Of these, 62 samples were paired: 24 pairs of subjects exposed to CPS and seven pairs exposed to sham stress. Clustering of the samples was evaluated through Multidimensional scaling plots (MDS) and a Permutational Multivariate Analysis of Variance (PERMANOVA) using the 10 calculated distances. We applied the adonis function from the mentioned Vegan R package to evaluate different variable effects in this clustering. We used as covariates the Time and the Patient and as a constraint the seqGroup (sequencing groups).

To identify taxonomic features (Phylum, Class, Order, Family, Genus and Species) that show significantly different abundances among the studied conditions, we used linear models, as implemented in the R package lme4 (v. 1.1-21) (Bates et al., 2015). A linear model was built (Supplementary Table S14) to assess the gender-dependent changes: the fixed effects that were considered were both the Time and the interaction of the Time and the Sex [tax_element ~ Time + Time:sex + (1| seqGroup)]. We indicated as a random effect the seqGroup variable.

We used ANOVA to assess the significance for each of the fixed effects included in the models using the Car R package (v. 3.0-6) (Fox et al., 2013). To assess particular differences between groups we performed multiple comparisons of the results obtained in the linear model using the multcomp R package's function glht and the Tukey test (v. 1.4-12) (Hothorn et al., 2008). To examine differences between groups considering interacting variables (Time and Sex) we used the lsmeans function from the lsmeans R package (v. 2.30-0) (Lenth, 2016). Due to the small sample size limiting the statistical power, no fdr correction was applied for multiple testing, but applied a bonferroni correction when comparing different groups. Statistical significance was defined when *P* values were lower than 0.05 in all the analyses.

Correlations among continuous variables were assessed using the cor.test function from the stats R package (v. 3.5-0). Normalization of the data was analyzed by using the shapiro.test function from the same package. The correlation coefficient (spearman or pearson) was specified taking into account the shapiro test result. In addition, a correlation matrix was performed for multiple comparisons using the corr.test function from the psych package (v. 2.0.12) (Revelle, 2023) and adjusting the *P*-values using the holm method.

When correlating FC after CPS of genes and other variables such as beta diversity metrics, some samples were removed because of missing data as indicated in the metadata (Supplementary Table S15).

## 3 Results

Forty-five subjects were initially recruited. Three were excluded from the study, of which two did not complete the protocol, and one had severe intraepithelial jejunal lymphocytosis after histological revision. In addition, five samples were excluded from the microbiome analysis due to poor DNA quality. A total of 37 participants were finally included, of which 30 underwent the CPS protocol (14 male, 16 female) and seven sham stress protocol (three male, four female; Figure 1). As mentioned, because of the quality of the sequencing data only 13 males and 11 females had pairing (pre and post) of the data. No significant differences were observed between the groups in key demographic or psychological parameters (stress and depression levels; Table 1 and Supplementary Table S1).

**Table 1:** Clinical variables from subjects submitted to cold pain stress.

	**Male**	**Female**	***P***
*n*	14	16	
Age (years)	22.8 (22.1–33.9)	22.9 (21.8–26.8)	0.759
Holmes-Rahe's score	98.5 (67.5–212.3)	69 (38.3–101.8)	0.058
Cohen'score	13.5 (10.5–22.5)	17 (11.5–21.5)	0.697
Beck's score	0 (0–2.5)	0 (0–1)	0.728
Menstrual phase (F/L)	N/A	7/8	

Data are expressed as median with first and third quartiles (Q1–Q3). A Mann–Whitney U-test was used for comparisons between groups.

F, follicular phase; L, luteal phase; N/A, not applicable.

### 3.1 Systemic response to CPS

#### 3.1.1 Autonomic response

**Figure 2:**
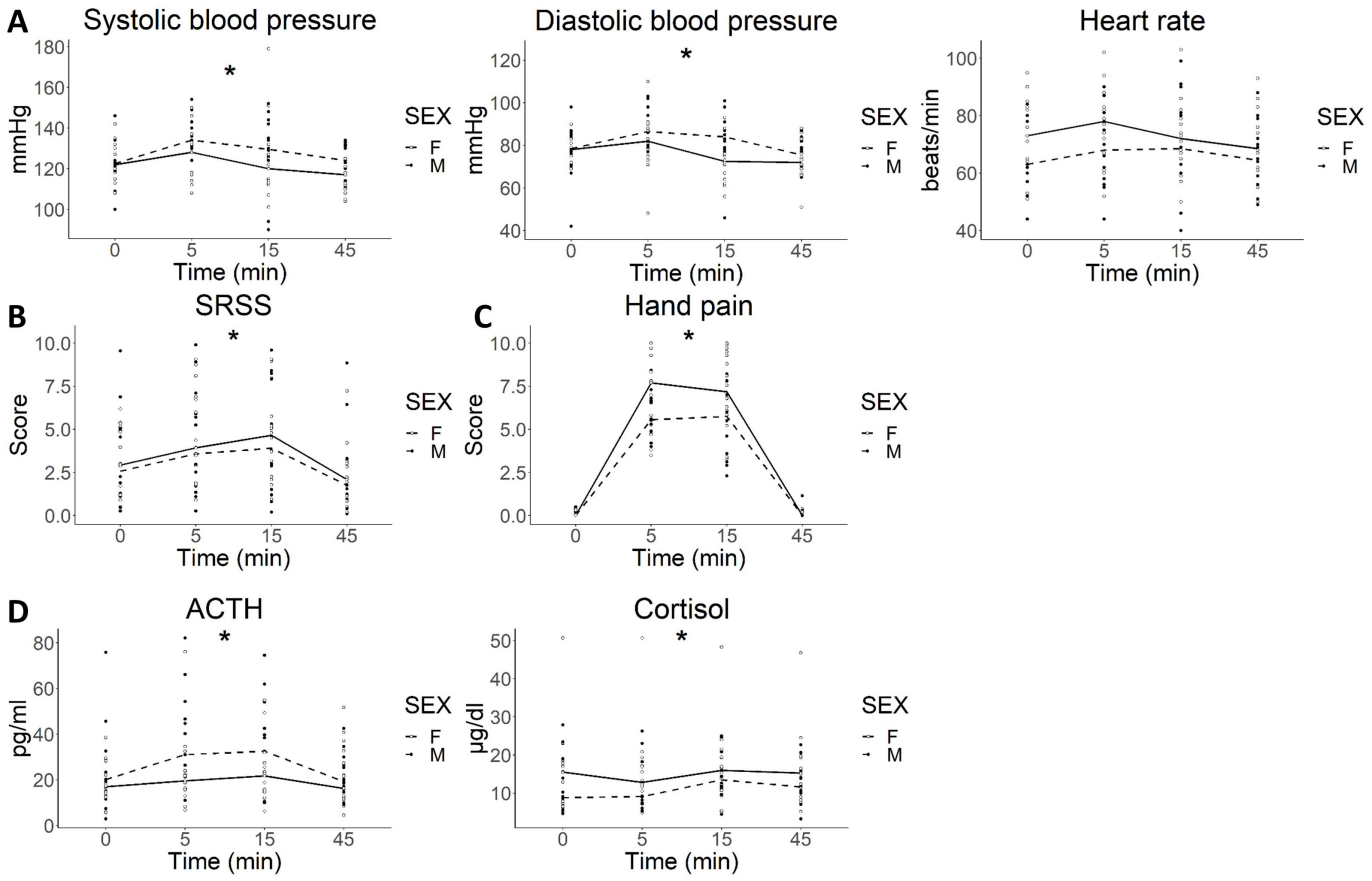
Systemic response to cold pain stress. **(A)** autonomic (blood pressure and heart rate); **(B)** psychological (SSRS); **(C)** hand pain and **(D)** hormonal responses to CPS. **(A)** Both systolic and diastolic blood pressure increased in response to stress (two-way ANOVA, ^*^P < 0.0001 for time) with no differences between sexes. **(B)** CPS significantly enhanced SSRS (two-way ANOVA, ^*^P < 0.0001), with no differences between sexes; **(C)** CPS was associated with a significant increase in hand pain perception (two-way ANOVA, ^*^P < 0.0001 for time) that was similar between sexes. **(D)** ACTH and cortisol levels significantly increased after CPS (two-way ANOVA, ACTH: ^*^P = 0.004; cortisol*: P* < 0.001 for time) with no differences by sex. Lines represent the median for each time point for M (dashed lines) and F (solid line). ACTH: adrenocorticotropic hormone; CPS: cold pain stress; min: minutes; SSRS: subjective stress rating scale.

CPS significantly increased systolic blood pressure (SBP) [*F*_(3, 83)_ = 6.450; *P* < 0.0001] and diastolic blood pressure (DBP) [*F*_(3, 83)_ = 8.210; *P* < 0.0001] in both groups, with no significant differences between men and women in SBP [*F*_(1, 27)_ = 1.481; *P* = 0.234] and DBP [*F*_(1, 27)_ = 2.348; *P* = 0.137; Figure 2A].

CPS was not associated with an increase in heart rate [HR; *F*_(3, 83)_ = 1.520; *P* = 0.215] (Figure 2A), and there were no differences between groups [*F*_(1, 27)_ = 1.825; *P* = 0.188].

Sham stress did not significantly increase SBP, DBP or HR in either group (Supplementary Figure S1).

#### 3.1.2 Psychological response

CPS was associated with an increase in the level of acute stress experienced by participants [*F*_(3, 76)_ = 8.972; *P* < 0.0001], which was similar between males and females [*F*_(1, 25)_ = 0,008; *P* = 0.931; Figure 2B].

Sham stress did not increase SSRS scores in either group (Supplementary Figure S1).

#### 3.1.3 Hand pain perception

CPS increased hand pain perception in both groups [*F*_(5, 140)_ = 109.766 *P* < 0.0001] with no differences between males and females [*F*_(1, 28)_ = 1.861; *P* = 0.183; Figure 2C].

#### 3.1.4 Hormonal response

CPS significantly increased plasma ACTH [*F*_(3, 78)_ = 4.747; *P* = 0.0043] and cortisol [*F*_(3, 83)_ = 6.778; *P* < 0.001], with no differences between males and females [*F*_(1, 26)_ = 2.281; *P* = 0.143] and [*F*_(1, 27)_ = 1.053; *P* = 0.314, respectively; Figure 2D].

Sham stress did not increase plasma ACTH or cortisol concentration in either group (Supplementary Figure S1).

### 3.2 Effect of CPS on the jejunal microbiota

The microbiome composition at baseline (PRE) and after CPS (POST) was dominated by the phyla *Firmicutes, Bacteroidetes, Proteobacteria and Fusobacteria* for both CPS and sham stress. The 10 most abundant genera were similarly distributed across all samples (Supplementary Figure S2).

**Figure 3:**
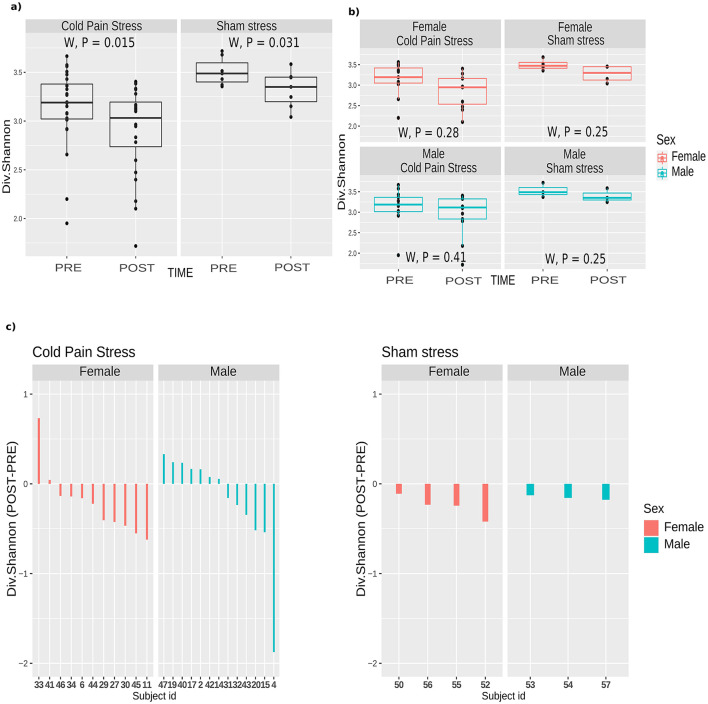
Small intestine mucosal microbiota response after cold pain or sham stress. Alpha diversity (Shannon index) changes in paired samples for both CPS (*n* = 48) and Sham stress (*n* = 14). **(A)** Shannon diversity according to the variable Time (PRE or POST). The Wilcoxon test (W) showed statistical significance (*P* = 0.015 and *P* = 0.031) for both CPS and Sham stress respectively. Boxplots of the Shannon index diversity are represented including the statistical significance. The line inside the boxplot represents the median for each of the group of samples **(B)** Shannon diversity according to Time and Sex. The Wilcoxon test showed no statistical significance (*P* = 0.28 and *P* = 0.41 for females and males respectively considering CPS, and *P* = 0.25 and *P* = 0.25 for females and males, respectively, considering sham stress). **(C)** Barplot of the difference of the Shannon diversity index (POST-PRE) in CPS and sham stress. The bars are colored according to the Sex. Samples are ordered by the magnitude of the Shannon index difference between their PRE and POST state.

CPS was associated with a significant decrease in alpha diversity (Figure 3A and Supplementary Figure S3). This decrease in the relative abundance of bacteria was more pronounced in females, although not significantly different from males (Figures 3B, C). We also observed a significant decrease in alpha diversity in the sham stress group (Figure 3A and Supplementary Figure S4). However, the differences were smaller compared to CPS subjects (Figure 3C).

**Figure 4:**
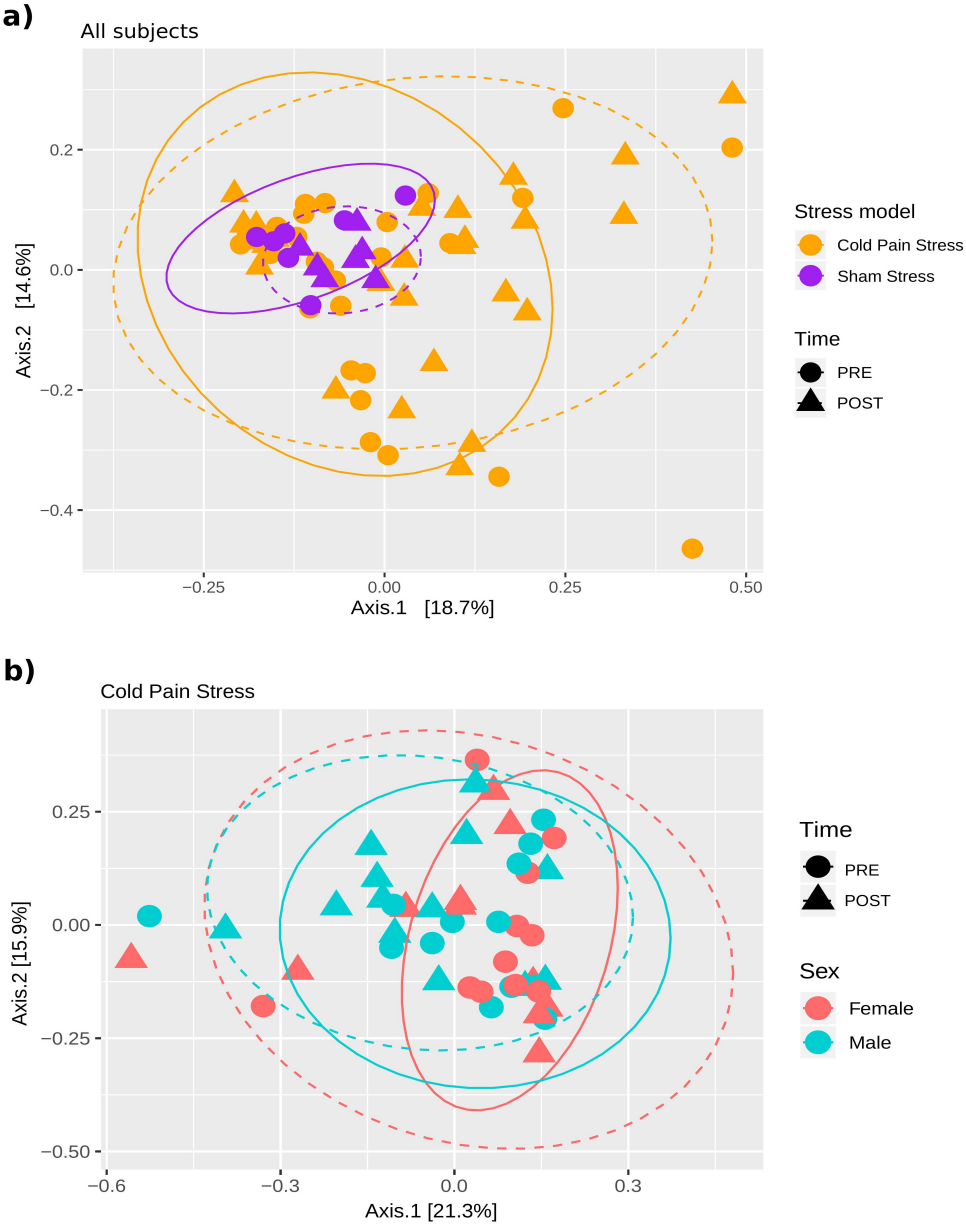
Effect of sex and stress on the small intestine mucosal microbiota. Multidimensional plot representing the Bray-Curtis dissimilarity. A significant effect of the Stress model (CPS or sham stress) and Sex was found in the clustering of the samples according to the Adonis test. Continuous and dashed ellipses are plotted for PRE and POST samples, respectively. **(A)** Samples are colored according to the Stress model and shaped according to Time. Significant effect of the Stress model (*n* = 68, Adonis test *P*-value: 0.001) **(B)** Samples are colored according to the Sex and shaped according to the Time. Significant effect of Sex (*n* = 48, Adonis test *P*-value: 0.013). Adonis test showed no statistically significant effect of the combination of time and Sex (*n* = 48, Adonis test *P* = 1).

We also assessed differences in the overall microbiota composition of the samples according to beta diversity metrics by assessing Multidimensional plots (MDS) of the Bray-Curtis dissimilarity of the samples, coloring and shaping them according to different variables, and assessing the statistical significance by the Adonis test. As observed in the MDS plot the sham stress samples showed relatively small differences between their baseline (PRE) or after sham stress (POST) biopsies, whereas the samples from individuals exposed to CPS varied more, especially among the POST samples (Figure 4A). Notably, we observed a greater similarity of PRE- and POST-stress samples in males than in females as shown by the overlap of the continuous and dashed ellipses (Figure 4B). Adonis test detected the Stress model as statistically significant effect variable on the overall composition, considering all the samples (*n* = 68, Adonis test *P*-value: 0.001) and the Sex when considering only paired cases, and including as covariate the individual id (*n* = 48, Adonis test *P*-value: 0.013).

### 3.3 Effect of sex in the CPS-induced changes in the jejunal microbiota

At baseline there were no significant differences between males and females in the relative abundances of bacteria.

**Figure 5:**
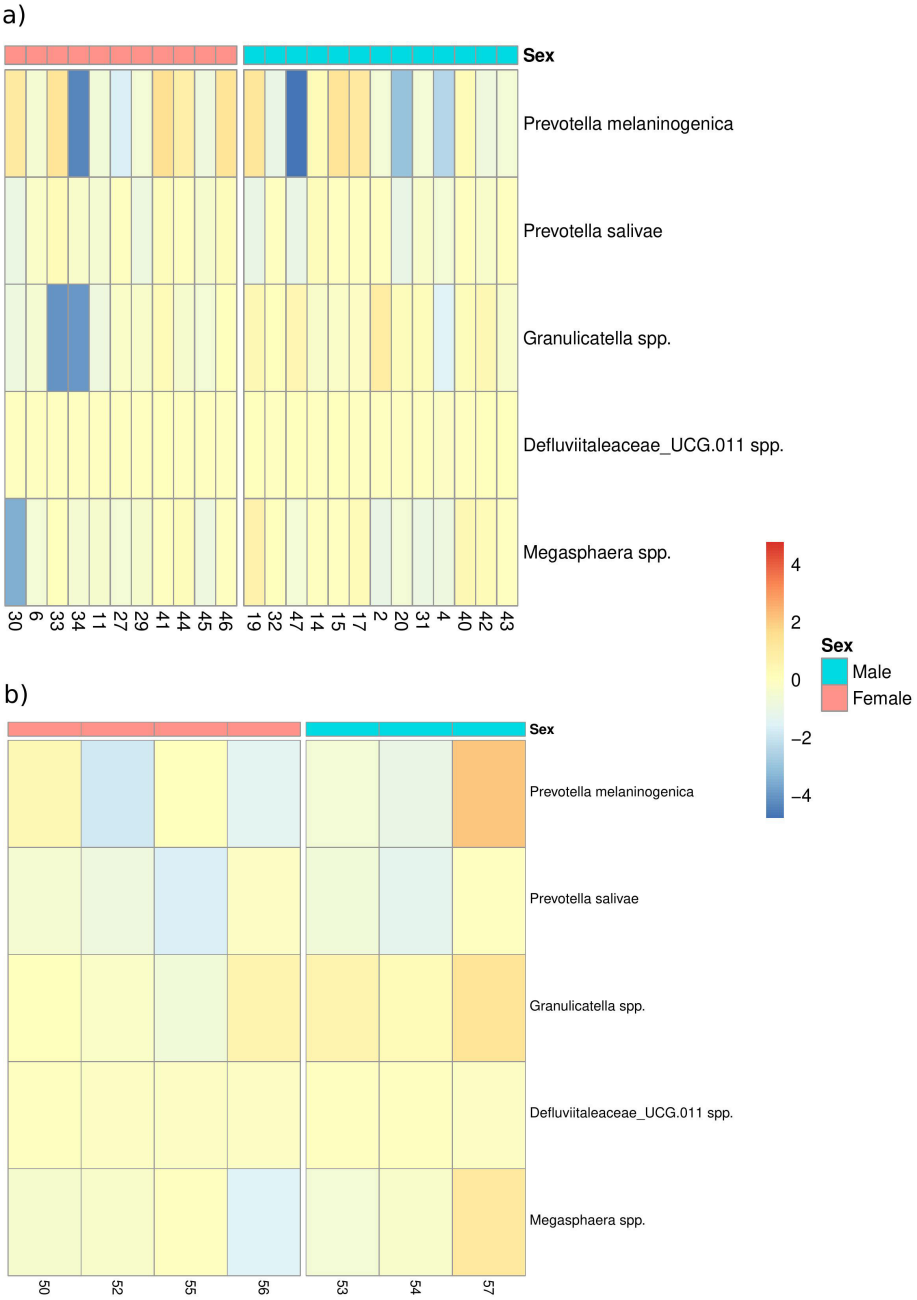
Difference in the taxa relative abundance after cold pain stress. Heatmap representation of the difference in the relative abundance multiplied by 100 of the taxa found as differentially abundant according to the *Time* considering both the *Time* and the interaction of the *Time* and the *Sex* as fixed effects (*n* = 48). Subject ids are depicted at *x*-axis. **(A)** Heatmap considering the CPS group (*n* = 48, cases). **(B)** Representation of these taxa in the sham group (*n* = 14, controls).

CPS was associated with significant differences in the relative abundance of several taxa at different taxonomic levels (Supplementary Table S2). In particular, five taxa were differentially abundant at the species level: *Prevotella melaninogenica, Prevotella salivae, Granulicatella* spp., *Defluviitaleaceae_UCG.011* spp., and *Megasphaera* spp. (Figure 5 and Supplementary Table S3). Interestingly, a different response was observed between males and females after the application of CPS, with 10 taxa having significant differential abundance (Supplementary Table S4).

### 3.4 Correlations between changes in the jejunal microbiota and molecular, hormonal and psychological responses to CPS

We investigated the relationship between changes in the jejunal microbiome and the expression levels of genes related to the epithelial barrier (*CLDN1, CLDN2, OCL, SLC26A3, TJP1*, and *TJP3*), stress regulation and circadian rhythm (*NR3C1, NR1D1, NR1D2, PER1*, and *PER3*), inflammation (*SOD1, IL18, NFE2L2, NIFL3*) and MC activation (*SERPINA1, TPSAB1*) from a previous expression analysis performed on the same intestinal samples (Rodiño-Janeiro et al., 2023) and the relationships of microbiome changes to participant's hormonal and psychological responses to stress.

Changes in the overall microbiome (beta diversity) significantly correlated with several gene expression changes (Fold Change, FC) after CPS (Supplementary Table S5). When stratifying by sex, we observed a significant negative correlation between OCL FC gene and beta diversity change only in the female group. In contrast, in the male group we observed eight significant correlations (Supplementary Tables S5, S6). Of note, we found a consistent negative correlation of the SOD1 gene FC and the change in total diversity of the samples only in males, suggesting that the less the change in microbial composition before and after the stress, the more the change in the expression levels of this particular gene.

**Figure 6:**
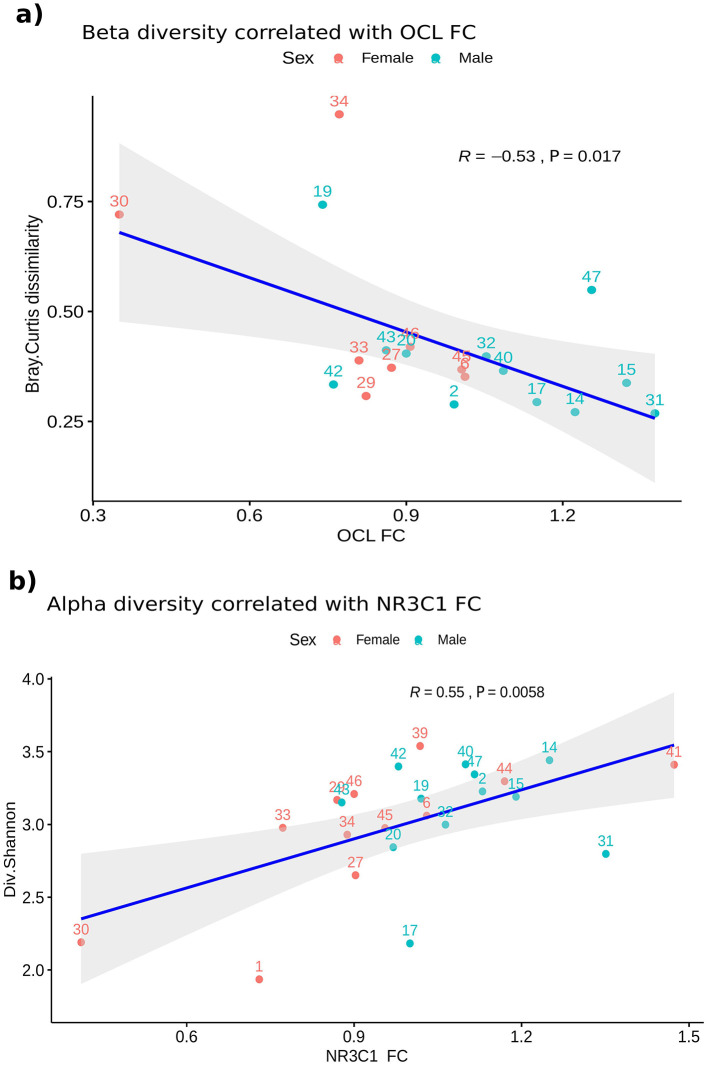
Correlation between changes in the small intestine mucosal microbiota and the expression levels of OCL and NR3C1. Scatterplots showing correlations of beta and alpha diversity variables and the FC of some genes (*n* = 22). Samples are colored according to the Sex. Both Rho (R) and *P*-value are represented in the plots. **(A)** Correlation between the Bray-Curtis dissimilarity of the samples before and after CPS and the FC of the OCL gene. Spearman test, Rho = −0.53 and *P* = 0.017. **(B)** Correlation between Shannon diversity and NR3C1 FC. Spearman test, Rho = 0.55 and *P* = 0.0058. CPS, cold pain stress; FC, fold change; NR3C1, glucocorticoid receptor nuclear receptor subfamily 3 group C member 1.

Alpha diversity variations also correlated with changes in the expression levels of some of the genes evaluated after CPS (Supplementary Table S7), as did the relative abundances of taxa after stress and the FC of the genes (Supplementary Table S8). Interestingly, a significant correlation was found between beta diversity and OCL FC after CPS, and between alpha diversity and NR3C1 FC after CPS, suggesting a relationship between gut microbiota and barrier-related gene expression (Figure 6).

We did not find a significant correlation between the overall change in the microbiome composition and cortisol or ACTH levels. However, significant correlations between specific taxa and stress hormones after CPS were observed (Supplementary Tables S9, S10).

Changes in psychological response to CPS, measured through the SSRS, were also correlated with differences in beta diversity (Supplementary Table S11). Stratifying by sex, we observed no significant correlation in females, but six significant correlations in males (Supplementary Table S12). Changes in microbiota composition that differed between males and females after CPS also correlated with changes in SSRS (Supplementary Table S13).

No significant correlation between the overall change in the microbiome composition and levels of life stress and depression, individuals' weight, height or body mass index were found.

## 4 Discussion

This study shows that acute stress causes rapid and considerable changes in the composition of jejunal microbiota. These changes are more evident in women, despite the absence of any differences in the systemic response to stress between the sexes. Changes in the composition of small intestine microbiota can contribute to IB dysfunction induced by stress through the brain-gut axis (BGA). This phenomenon may account for the higher risk of developing DGBI in women. Disruption of this network can lead to impaired GI function, and has been associated with inflammatory bowel disease (IBD) and IBS.

Gut microbiological changes can be induced by enteric infections, antibiotics, and stress. Pediatric patients with Crohn's disease (CD) (Mackner et al., 2020) and pregnant women with high anxiety/stress (Hechler et al., 2019) display differences in the fecal microbiome and metabolome, indicating increased levels of inflammation, with higher relative abundance of *Proteobacteria* and lower relative abundance of lactic acid bacteria and *Bifidobacteria* (Zijlmans et al., 2015). Changes in gut microbiota composition during pregnancy can be associated with several childhood adversities, which may contribute to altered inflammatory and glucocorticoid responses to stress (Hantsoo et al., 2019). There is evidence that mucosal microbiota composition can rapidly change after acute stress (Lyte and Ernst, 1992; Zhang et al., 2021, 2024). This study shows novel information suggesting that acute stress rapidly alters jejunal bacterial composition in healthy subjects, with more pronounced changes observed in females. Importantly, these differences are not related to chronic stress since there were no significant differences in chronic stress levels between male and female groups.

Sex-related changes in the gut microbiota have been previously reported in animal models of early life stress (Park et al., 2021), after chronic stress (Lyte et al., 2020, 2022), and in response to NSAIDs in humans (Haro et al., 2016; Edogawa et al., 2018). Patients with functional GI symptoms and IBD have shown lower phylogenetic alpha diversity, richness, and evenness with significant decreases in the genera *Porphyromonas, Prevotella*, and *Fusobacterium*, when compared to healthy subjects (Saffouri et al., 2019; Simrén et al., 2013; Ni et al., 2017). In this study, we also found a significant sex-dependent decrease in an unclassified species of the genus *Alloprevotella*, which was previously reported to be increased in a male mouse model of psychosocial stress (Burokas et al., 2017). We found a significant decrease in the species *Rothia mucilaginosa* species in females after CPS, a taxon that has been associated with oral contraceptive use (Sinha et al., 2019) and shown to be decreased in CD (Gevers et al., 2014). Dysregulation of *Granulicatella* spp., which we also found to be decreased after CPS, has also been described in CD after ileocolic resection (Mondot et al., 2016).

Stress, microbiota, and IB function are closely interlinked. Changes in the microbiota have been observed in healthy individuals following a multi-stressor military training environment, which also increased gut permeability (Karl et al., 2017). This is also seen in patients with alcohol abuse where richness and evenness of the fecal microbiota were reduced along with increased gut permeability (Maccioni et al., 2020). In mice subjected to water avoidance stress, psychological stress exacerbates NSAID-induced small bowel injury through changes in intestinal microbiota and barrier permeability induced by glucocorticoid receptor signaling (Yoshikawa et al., 2017). Indeed, the tight junction protein Claudin-1 promoter is regulated by the glucocorticoid receptor and the transcriptional repressor HES1 (Zheng et al., 2017). This suggests that NR3C1 is responsible for regulating the stress-induced decrease in intestinal permeability. After CPS, significant correlations were found between changes in gene expression and alpha and beta diversity metrics in genes related to inflammation, epithelial barrier, MC activation, circadian rhythm and stress response. Our results indicate a positive correlation between alpha-diversity and NR3C1 FC, and a negative correlation between beta-diversity and SOD1 and OCL. Beta-diversity negatively correlated with NR3C1 FC in males. In contrast, alpha diversity positively correlated with NR3C1 and CLDN1 while beta diversity positively correlated with OCL FC in females. These results suggest that changes in the microbiota are associated with changes in the expression of genes encoding different proteins related to gut barrier. Additionally, significant correlations were observed between psychological and hormonal responses to CPS and beta diversity overall changes in intestinal microbiota. This implies that psychological stress modulates changes in intestinal microbiota through BGA.

The mechanisms responsible for the rapid changes observed *in* the microbiota are not yet fully understood. although previous studies have described some of them. According to Rengarajan et al. (2020), stress induces fecal dysbiosis, IB dysfunction, and antibacterial IgA release in mice. In this context, we have previously demonstrated an increased humoral immune response in IBS-D patients, although it is more linked to IgG than to IgA (Vicario et al., 2015). Moreover, a study has proved that female microbiota transfer to germ-free mice leads to significantly lower IgA levels compared to male microbiota transfer (Fransen et al., 2017). Luminal levels of IgA were not assessed, but changes in salivary IgA secretion by stress reach peak concentration in 10 minutes (Seizer et al., 2024) being a response that could explain the observed changes in gut microbiota after CPS. According to recent research, chronic social defeat stress significantly reduces alpha-defensin secretion and induces dysbiosis, which can be reversed in mice by administering alpha-defensin (Suzuki et al., 2021). In contrast, we discovered an increase in luminal alpha-defensin secretion in the jejunum of healthy women after acute stress (Alonso et al., 2008, 2012; Vanuytsel et al., 2014) being secreted by CPS in 15 min (Alonso et al., 2012). However, although the rapid response matches the altered microbiota timeline, further studies are required to understand the impact of stress on the regulation of alpha-defensins and the small bowel microbiota. Lastly, CPS increases small bowel water secretion in 15 minutes (Alonso et al., 2008, 2012), a mechanism that appears to have a relevant influence the colonization by pathobionts and the stability of the intestinal microbiota (Rengarajan et al., 2020) and that was more pronounced in female subjects than male subjects (Alonso et al., 2012). Therefore, both innate and adaptive immune responses can contribute to modulate intestinal microbiota, and recent studies have highlighted the sensitivity of the upper gastrointestinal tract to these immune-modulated changes (Gu et al., 2022). Also, significant sex differences in gut immune system have been described (Sankaran-Walters et al., 2013) that could affect the types and abundance of microbial communities, potentially altering beta-diversity between male and females.

Our study has several limitations related to the complexity, of the methodology that could act as confounding factors for the interpretation of our results, including a relatively small sample size, particularly in the sham stress group, a distinct anticipatory and stress response to baseline biopsy between groups, a slight difference in chronic stress levels between males and females in the CPS group that could affect jejunal response, no negative controls to account for potential contamination, and the inability to test intestinal permeability in parallel with the biopsy procedure.. Moreover, the decision to choose the timing of the second biopsy (90 min after CPS) was arbitrary, and we recognize that more pronounced changes could occur earlier or later. Furthermore, we acknowledge the possibility that the observed changes are a result of the experimental procedure's combined effect, including stress related to intubation and baseline biopsy, as well as to sham/CPS, given that microbial changes were also found following the sham stress protocol.

However, our study also has important strengths. There are limited studies that have examined the composition of human microbiota in the small intestine, an area that has received considerably less attention compared to the large intestine in existing studies and even fewer have investigated the impact of sex differences and stress response *in vivo*. A recent systematic analysis of saliva, mucosal, and fecal samples from healthy subjects showed that the GI tract can be categorized into four unique regions based on their bacterial composition, and indicated that the fecal microbiota does not accurately represent the mucosal microbiota (Vasapolli et al., 2019). Here, we report the changes in the jejunal mucosal microbiota induced by stress in a larger sample, despite the challenges in accessing this segment. Although one small biopsy is probably not enough to give proper indication about the microbial composition as there is evidence that the mucosal surface is not homogeneous throughout the gastrointestinal tract, and differences in microbiota composition have been shown both at the mucosal level (Vasapolli et al., 2019; Seekatz et al., 2019; Li et al., 2016), and at the intestinal lumen (An et al., 2024), intestinal biopsies were taken from the same place and all participants underwent the same experimental procedure, supporting the credibility of our observations. These changes are believed to have a significant impact on host immunity (Van den Abbeele et al., 2011; Uchimura et al., 2018), and the regulation of digestion (Martinez-Guryn et al., 2018), and could potentially contribute to the pathophysiology of IBS.

In summary, acute stress swiftly changes the gut microbiota's composition in the jejunum of healthy individuals. Women show more pronounced changes than men, including some female-specific changes in certain species. If further validated, our results can aid in understanding the female predominance in DGBI. However, further research with larger cohorts is required to confirm and expand these findings in order to potentially identify specific microbial signatures that may predict BGA susceptibility to stress-sensitive disorders.

## Data Availability

The data that support the findings of this study (16S rRNA gene sequencing raw sequences) can be accessed in Sequence Read Archive database with accession code PRJNA807111.
