# Supplementary material for: Acute stress triggers sex-dependent rapid alterations in the human small intestine microbiota composition
Source: Front Microbiol. 2025 Jan 15;15:1441126. doi: 10.3389/fmicb.2024.1441126 (PMC11778178; doi:10.3389/fmicb.2024.1441126)
Supplement: Supplementary file 1 [file Data_Sheet_1.pdf]

SUPPLEMENTARY MATERIAL

RESULTS

Systemic response to Sham stress

Autonomic response

Sham stress was associated with a significant decrease in SBP [F(5,15)=4.777; *P=0.016*], which differed between male and females [F(1,5 =7.089; *P*=0.045] (Figure S1A), but did not affect DBP [F(3,15)=2.412; *P=0.107*], which did not differ between males and females [F(1,5=0.275; *P*=0.622] (Figure S1A).

Sham stress was also associated with a significant decrease in HR [F(3, 15) =3.440; *P=0.044*], with no differences between groups F(1,5=5.031; *P*=0.0749] (Figure S1A).

Psychological response

Sham stress did not increase the levels of acute stress experienced by the participants [F(3, 15) =0.947; *P=0.443*], and there were no differences between groups [F(1, 5) = 0,015; *P*=0.909] (Figure S1B).

Hormonal response

Sham stress was associated with a decrease in plasma ACTH [F(3,15) = 8.50; *P=0.002*] that differed between sex groups [F(1,5) =10.99; *P=0.021*] and with a decrease in plasma cortisol [F(3, 15) = 20.412; *P<0.0001*] that was different between sex groups [F(1,5) =12.72; *P=0.016*] (Figure S1C).

TABLES

Table S1. Clinical variables from subjects submitted to sham stress.

	**Male**	**Female**	**P. value**
**n**	3	4	
**Age (years)**	26.1 (20.4.2-30.2)	24.3 (21.8-32.4)	0.64
**Holmes-Rahe's score**	45 (41-158)	100 (60-130.5)	0.13
**Cohen'score**	14 (11-20)	9.5 (6.5-19)	0.32
**Beck's score**	0 (0-1.8)	0 (0-3)	0.67
**Menstrual Phase (F/L)**	N/A	2/2	

Data are expressed as median with first and third quartiles Q1–Q3). A Mann–Whitney U test was used for comparisons between groups. F: follicular phase; L: luteal phase; N/A: not applicable.

Table S2. Effect of sex in the CPS-induced changes in the jejunal microbiome. Summary of the significant differential abundance analysis results applying a linear model including all paired cases (n=48). Fixed effects: Time and the interaction of the Time and the Sex (Time:sex). Random effect: seqGroup.

	Phylum	Class	Order	Family	Genus	Species
**Time**	0	1	1	2	3	5
**Time:sex**	1	0	3	5	8	10

Table S3. Average relative abundance of the taxa found as differentially abundant after sham stress and CPS.

Species	CPS PRE %	CPS POST %	Sham stress PRE %	Sham Stress POST %	P.Value
Prevotella melaninogenica	2.00	1.51	1.59	1.32	0.046
Prevotella salivae	0.49	0.21	0.79	0.19	0.032
Granulicatella spp.	0.92	0.48	0.62	0.85	0.025
Defluviitaleaceae_UCG.011 spp.	0.005	0.02	0.03	0	0.026
Megasphaera spp.	0.74	0.38	0.89	0.66	0.031

Table S4: Average relative abundance of the taxa found as differentially abundant after CPS between sex groups.

Species	CPS PRE Females %	CPS POST Females %	CPS PRE Males %	CPS POST Males %	P.Value
Actinomyces odontolyticus	0.4444	0	0.0464	0.1278	0.041
Gemella sanguinis	0.1517	0.0536	0	0.0135	0.045
Megasphaera micronuciformis	0	0.2353	0.3487	0.1472	0.026
Rothia mucilaginosa	0.5323	0.0984	0.0316	0.2098	0.009
Brevibacterium spp.	0	0.0040	0.0248	0	0.049
Alloprevotella spp.	3.4561	2.3266	5.7173	6.0334	0.022
Bergeyella spp.	0.2111	0.1816	1.6612	0.9319	0.030
Granulicatella spp.	1.3684	0.3562	0.5374	0.5834	0.007
F.Family_XIII.UCG	0.1951	0.1049	0.0627	0.0808	0.042
Megasphaera spp.	1.0869	0.5005	0.4527	0.2863	0.016

Table S5. Significant correlations between the FC of the studied genes and the differences between samples measured by distance metrics after CPS. Both sexes are considered (n=22, Only paired data was taken into account because of the calculation of beta diversity metrics between pre and post of each of the individuals. Individual #4 (Male) and #11 (Female) were removed because not FC data was available for them).

**SPEARMAN TEST:**			
**Gene**	**Beta diversity distance**	**Rho/Cor**	**P-value**
SOD1	Bray-Curtis	-0.4997	0.0179
SOD1	Jaccard	-0.4908	0.0204
OCL	Bray-Curtis	-0.5278	0.0168
NR1D2	Jaccard	-0.4524	0.0345
NR1D2	Canberra	-0.4545	0.0336
NFE2L2	Jaccard	-0.4495	0.0358
**PEARSON TEST:**			
**Gene**	**Beta diversity distance**	**Rho/Cor**	**P-value**
SOD1	Canberra	-0.5648	0.0062
SOD1	Gunia05	-0.5774	0.0049
OCL	Canberra	-0.4489	0.0471

CPS: cold pain stress; SOD1: Superoxide dismutase 1; OCL: occludin; NR1D2: Nuclear Receptor Subfamily 1 Group D Member 2; NFE2L2: Nuclear factor erythroid 2-related factor 2.

Table S6. Significant correlations between the FC of the studied genes and the differences between samples measured by distance and dissimilarity metrics after CPS in males (n=12, Male individual #4 not considered as it had no FC genes data).

**SPEARMAN TEST:**			
**Gene**	**Beta diversity distance**	**Rho/Cor**	**P-value**
SOD1 FOLD	Bray-Curtis	-0.6084	0.0358
SOD1 FOLD	Aitchison	-0.6364	0.0261
NR1D2 FOLD	Jaccard	-0.6491	0.0224
NR1D2 FOLD	Canberra	-0.6853	0.0139
**PEARSON TEST:**			
**Gene**	**Beta diversity distance**	**Rho/Cor**	**P-value**
SOD1 FOLD	Jaccard	-0.8159	0.0012
SOD1 FOLD	Canberra	-0.8129	0.0013
SOD1 FOLD	GuniVAW	-0.5871	0.0447
SOD1 FOLD	Gunia05	-0.7633	0.0039
NR3C1 FOLD	Gunia05	-0.6586	0.0199

CPS: cold pain stress; SOD1: Superoxide dismutase 1; NR1D2: Nuclear Receptor Subfamily 1 Group D Member 2; NR3C1: glucocorticoid receptor nuclear receptor subfamily 3 group C member 1.

Table S7. Significant correlations between the FC of the studied genes and the alpha diversity metrics after CPS. Both sexes are considered (n=26, Including all cases at CPS status (not only paired data)).

**SPEARMAN TEST:**			
**Gene**	**Alpha diversity measure**	**Rho/Cor**	**P-value**
IL18	Div.Shannon	0.4826	0.0169
NIFL3	Div.Simpson	0.4626	0.0228
NIFL3	Faiths.PD	-0.4426	0.0303
NR1D2	Div.Shannon	0.42	0.0410
PER3	Div.Shannon	0.4339	0.0341
PER3	Faiths.PD	0.4183	0.0420
NFE2L2	Div.Shannon	0.4200	0.0410
TRYP	Gene_counts	0.4643	0.0223
NR3C1	Div.Shannon	0.5461	0.0058
SERPINA1	Div.Shannon	0.4078	0.0479
**PEARSON TEST:**			
**Gene**	**Alpha diversity measure**	**Rho/Cor**	**P-value**
NIFL3	Div.InvSimpson	0.4400	0.0314
NR3C1	Div.InvSimpson	0.4864	0.0159

CPS. Cold pain stress; IL18: interleukin-18; NIFL3: Nuclear Factor, Interleukin 3 Regulated; NR1D2: Nuclear Receptor Subfamily 1 Group D Member 2; PER3: period circadian protein homolog 3; NFE2L2: Nuclear factor erythroid 2-related factor 2; TRYP: tryptase; NR3C1: glucocorticoid receptor nuclear receptor subfamily 3 group C member 1; SERPINA1: serpin family A member 1.

Table S8. Significant correlations between the FC of the studied genes and taxa relative abundances after CPS. Results containing only Rho or Cor greater than 0.5 or lower than -0.5. Both sexes are considered (n=26, Including all cases at CPS status (not only paired data)).

SSPEARMAN TEST:			
**Gene**	**Taxa relative abundances after CPS**	**Rho /Cor**	**P-value**
NR1D1 FOLD	*Campylobacter concisu*	0.5041	0.0120
NR1D1 FOLD	*Prevotella salivae*	0.5151	0.0100
PER1 FOLD	*Aggregatibacter aphrophilus*	0.5158	0.0099
PER1 FOLD	*Lachnoanaerobaculum.cf* spp.	0.5579	0.0046
PER1 FOLD	F.Muribaculaceae.UCS	-0.5191	0.0093
IL18 FOLD	*Ruminococcaceae_UCG.014* spp.	0.5511	0.0053
SOD1 FOLD	*Solobacterium* spp.	0.5416	0.0063
CLDN1 FOLD	*Alloprevotella rava*	-0.5326	0.0107
CLDN1 FOLD	*Johnsonella* spp.	0.5391	0.0096
CLDN1 FOLD	*Peptococcus* spp.	0.5103	0.0153
CLDN2 FOLD	*Filifactor alocis*	0.5068	0.0115
OCL FOLD	*Corynebacterium* spp.	-0.5473	0.0084
TJP1 FOLD	*Campylobacter concisus*	0.5595	0.0045
TJP1 FOLD	*Neisseria flavescens*	0.5705	0.0034
TJP1 FOLD	*Lactobacillus* spp.	-0.5781	0.0031

TJP1 FOLD	*Escherichia* spp.	-0.5038	0.0121
NIFL3 FOLD	*Lautropia mirabilis*	-0.5370	0.0068
NR1D2 FOLD	*Ruminococcaceae_UCG.014 spp.*	0.5109	0.0107
PER3 FOLD	*Dialister spp.*	0.5399	0.0065
TJP3 FOLD	*Campylobacter concisu*	0.5776	0.0031
TJP3 FOLD	*Lachnoanaerobaculum.cf* spp.	0.5599	0.0044
TJP3 FOLD	*Atopobium* spp.	0.5285	0.0079
TJP3 FOLD	F.Muribaculaceae.UCS	-0.5103	0.0108
TJP3 FOLD	*Lactobacillus* spp.	-0.5669	0.0037
TJP3 FOLD	*Solobacterium* spp.	0.5399	0.0065
TRYP FOLD	*Cloacibacterium normanense*	-0.5339	0.0072
TRYP FOLD	*Rubrobacter* spp.	-0.5575	0.0046
TRYP FOLD	*Capnocytophaga* spp.	0.5048	0.0119
TRYP FOLD	*Neisseria* spp.	0.5278	0.0080
NR3C1 FOLD	*Ruminococcaceae_UCG.014* spp.	0.5607	0.0044
NR3C1 FOLD	*Solobacterium* spp.	0.5015	0.0125
NR3C1 FOLD	F.Saccharimonadaceae.UCS	0.5048	0.0119
SERPINA1 FOLD	*Stomatobaculum longum*	0.5144	0.0101

SERPINA1 FOLD	*Lactobacillus* spp.	-0.5743	0.003
SERPINA1 FOLD	*Solobacterium* spp.	0.5481	0.0055
PER1 FOLD	*Staphylococcus* spp.	-0.6008	0.0019
CLDN2 FOLD	*Stomatobaculum longum*	0.6173	0.0013
OCL FOLD	*Lactobacillus* spp.	-0.6082	0.0027
TRYP FOLD	*Campylobacter* spp.	0.6092	0.0016
SERPINA1 FOLD	*Lachnoanaerobaculum.cf* spp.	0.7068	0.0001
SERPINA1 FOLD	O.Chloroplast.UCF	-0.6277	0.0010
SERPINA1 FOLD	F.Saccharimonadaceae.UCG	0.6057	0.0017
**PEARSON TEST:**			
**Gene**	**Taxa relative abundances after CPS**	**Rho /Cor**	**P-value**
NR3C1 FOLD	*Veillonella* spp.	0.5678	0.0038
SERPINA1 FOLD	*Veillonella* spp.	0.5234	0.0087

CPS: cold pain stress; NR1D1: Nuclear Receptor Subfamily 1 Group D Member 1; PER1: period circadian protein homolog 1; IL18: interleukin-18; SOD1: Superoxide dismutase 1; CLDN1: claudin 1; CLDN2: claudin 2; OCL: occludin; TJP1 FOLD: tight junction protein-1; NIFL3: Nuclear Factor, Interleukin 3 Regulated; NR1D2: Nuclear Receptor Subfamily 1 Group D Member 2; PER3: period circadian protein homolog 3; TJP3: tight junction protein-3; TRYP: tryptase; NR3C1: glucocorticoid receptor nuclear receptor subfamily 3 group C member 1; SERPINA1: serpin family A member 1.

Table S9. Significant correlations between taxa relative abundances and hormones (Cortisol and ACTH) after CPS (n=26, Including all cases at CPS status (not only paired data)). Results containing only Rho greater than 0.5 or lower than -0.5. Both sexes are considered.

Final taxa relative abundances	Final hormone	Rho	P-value
Helicobacter pylori	Cortisol_T105	-0.5339	0.0072
Porphyromonas spp.	ACTH_T105	0.5158	0.0099
Capnocytophaga spp.	ACTH_T105	0.5651	0.0040
Helicobacter spp.	Cortisol_T105	-0.5039	0.0121
Defluviitaleaceae_UCG.011 spp.	ACTH_T105	0.6502	0.0006
Catonella spp.	ACTH_T105	0.5166	0.0097
Stenotrophomonas spp.	Cortisol_T105	0.5310	0.0076
Treponema spp.	ACTH_T105	0.6139	0.0014

ACTH: adrenocorticotropic hormone; CPS: cold pain stress.

Table S10. Significant correlations between change of taxa relative abundances (T105 - T-65) and hormone changes after CPS (T105 – T-65) (n=24, Including only paired data). Results containing only Rho or Cor greater than 0.5 or lower than -0.5.

**SPEARMAN TEST:**			
**Change in taxa relative abundances**	**Change in hormones**	**Rho / Cor**	**P-value**
Lachnoanaerobaculum.cf spp.	Change_ACTH	-0.5114	0.0126
Family_XIII_UCG.001 spp.	Change_ACTH	-0.5856	0.0033
Catonella spp.	Change_ACTH	-0.6123	0.0019
Paracoccus spp.	Change_cortisol	-0.5604	0.0054
Diaphorobacter spp.	Change_ACTH	-0.6734	0.0004
Stenotrophomonas spp.	Change_cortisol	-0.6152	0.0018
**PEARSON TEST:**			
**Change in taxa relative abundances**	**Change in hormones**	**Rho / Cor**	**P-value**
Peptostreptococcus stomatis	Change_ACTH	-0.5176	0.0114
Parvimonas spp.	Change_ACTH	-0.5117	0.0126
F.Saccharimonadaceae.UCG	Change_cortisol	-0.5039	0.0142

ACTH: adrenocorticotropic hormone.; CPS: cold pain stress.

Table S11. Significant correlations between the beta diversity and SSRS after CPS. Both sexes are considered (n=24 , Including only paired data).

**Beta diversity distance**	**SSRS interval**	**Rho**	**P-value**
Jsd	T15_10	0.5919	0.0258
Bray-Curtis	T15_10	0.6271	0.0164
Jaccard	T0_-95	-0.6818	0.0026
Aitchison	T15_10	0.5765	0.0309

CPS: cold pain stress; SSRS: subjective stress rating scale.

Table S12. **Significant correlations between the beta diversity and SSRS after CPS in males**. Only males are considered (n=13, Including only paired males).

**SPEARMAN TEST:**			
**Beta diversity distance**	**SSRS interval**	**Rho / Cor**	**P-value**
Bray-Curtis	T105_45	0.6455	0.037
**PEARSON TEST:**			
**Beta diversity distance**	**SSRS interval**	**Rho / Cor**	**P-value**
Jsd	T10_5	-0.9344	0.0063
Bray-Curtis	T10_5	-0.9072	0.0125
Canberra	T10_5	-0.8937	0.0163
Canberra	T105_45	0.6562	0.0283
Gunia05	T105_45	0.6232	0.0405

CPS: cold pain stress; SSRS: subjective stress rating scale.

Table S13. Significant correlations between SSRS and changes in relative abundances of differentially abundant taxa after CPS. (n=24, Including only paired data).

SSRS interval	Change in Taxa relative abundance	Rho	P-value
T0_-95	*Megasphaera* spp.	-0.5270	0.0297
T10_5	*Actinomyces odontolyticus*	-0.5452	0.0438
T10_5	*Megasphaera micronuciformis*	0.7764	0.0011
T15_10	*Rothia mucilaginosa*	-0.5397	0.0464
T20_15	*Rothia mucilaginosa*	0.4739	0.0348
T20_15	T20_15_Stress~*Granulicatella* spp.	0.5197	0.0188
T20_15	*F.Family_XIII.UCG*	0.6281	0.0030

CPS: cold pain stress; SSRS: subjective stress rating scale.

Table S14. **Variables specified in the linear model used for the differential analyses.**

Name linear model	Fixed effects		Random effects
	**Variable**	**Labels**	
Time:Sex	Time	PRE or POST	seqGroup (sequencing run)
	Time:Sex	PRE or POST: Females or Males	

The name of the linear model indicates the main fixed effect that it considers. For each fixed effect we indicate the associated labels, with the following meanings: PRE: Before CPS; POST: After CPS; "Time:Sex" denotes a variable that takes into account the interaction between the Time and Sex variables. SeqGroup: 2018_only, 2018_reseq, 2019_only or 2019_reseq.

Figure Legends

Figure S1: Systemic response to sham stress protocol. A) autonomic (blood pressure and heart rate); B) psychological (SSRS); and C) hormonal responses during sham stress. (A) Systolic blood pressure significantly decreased during sham stress (two-way ANOVA, **P*=0.016 for time), with differences between M and F (*P*=0.045); heart rate significantly decreased during sham stress (two-way ANOVA, * *P*=0.044 for time), with no differences between M and F. (B) Sham stress did not significantly modify subjective stress rating scale (SSRS) (two-way ANOVA, *P*=0.443); (C) Sham stress was associated with a significant decrease in ACTH that was different between M and F (two-way ANOVA, **P*=0.002 for time, *P*=0.021 for sex); cortisol levels significantly decreased during sham stress, with no differences between M and F (two-way ANOVA, * *P*<0.0001). Lines represent the median for each time point for M (dashed lines) and F (solid line). ACTH: adrenocorticotropic hormone; F: females; M: males; min: minutes; SSRS: subjective stress rating scale.

Figure S2. Average relative abundance of each of the top 10 genera according to *Time* (PRE or POST) considering the two stress models (Cold pain stress and Sham stress) and the SexLinar models including the Time, Stress model and Sex as fixed effects and the Sequencing batch and individual id as random effects detected Prevotella (P= 0.006) and Actinobacillus (P=0.014) as the only significantly differential abundant genus according to Time, considering as covariates the rest of variables.

Figure S3. Alpha diversity characterization after CPS. Boxplots of the different diversity indices are represented including the statistical significance. The line inside the boxplot represents the median for each of the group of samples. Observed and Simpson indices according to the variable *Time* (PRE or POST). The Wilcoxon test showed statistical significance in Simpson index (n= 48, P = 0.016). CPS: cold pain stress.

Figure S4. Alpha diversity after sham stress. Boxplots of the different indices are represented including the statistical significance. Observed and Simpson indices according to the *Time.* The Wilcoxon test showed statistical significance in the Observed index (n=14, P = 0.036).
