# Supplementary figures and images for: Acute stress triggers sex-dependent rapid alterations in the human small intestine microbiota composition
Source: Front Microbiol. 2025 Jan 15;15:1441126. doi: 10.3389/fmicb.2024.1441126 (PMC11778178; doi:10.3389/fmicb.2024.1441126)

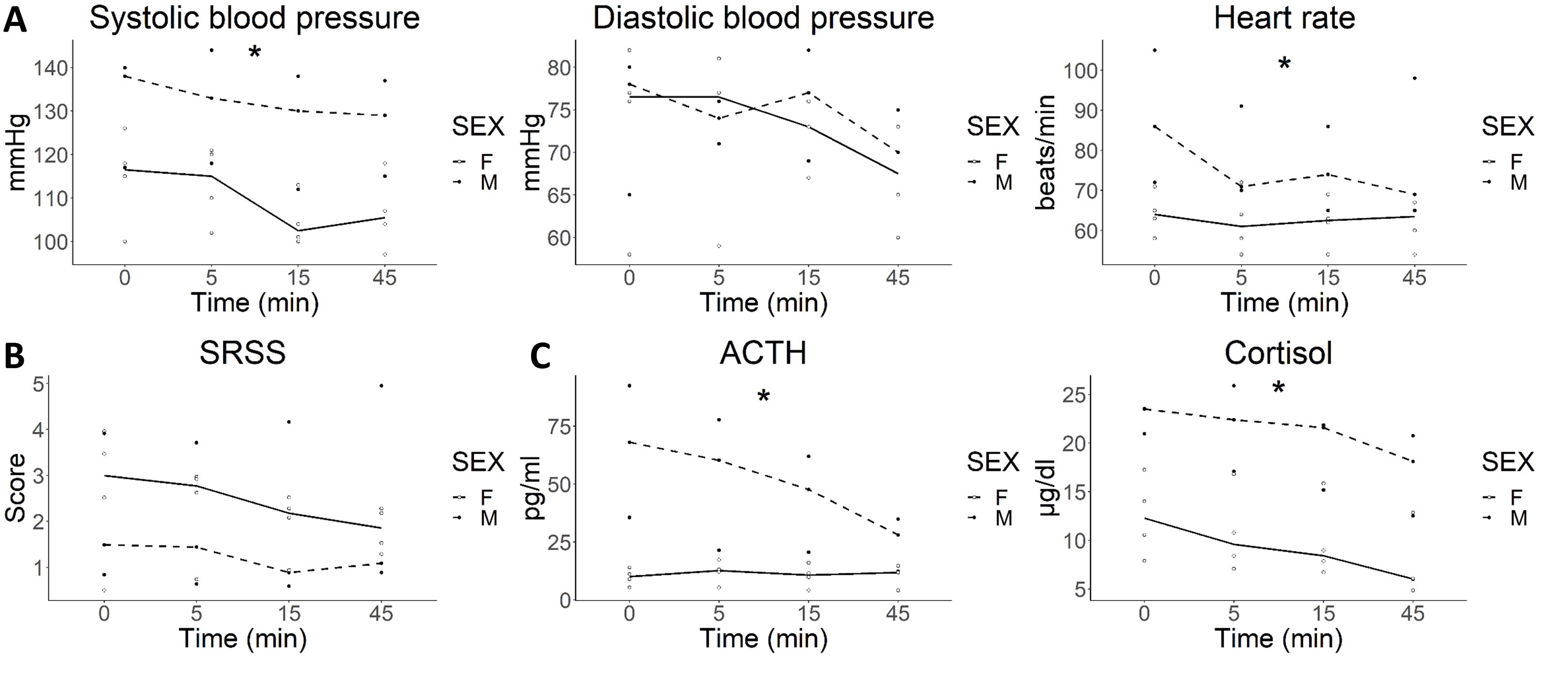

Supplement: Supplementary file 3 [file Image_1.jpeg]

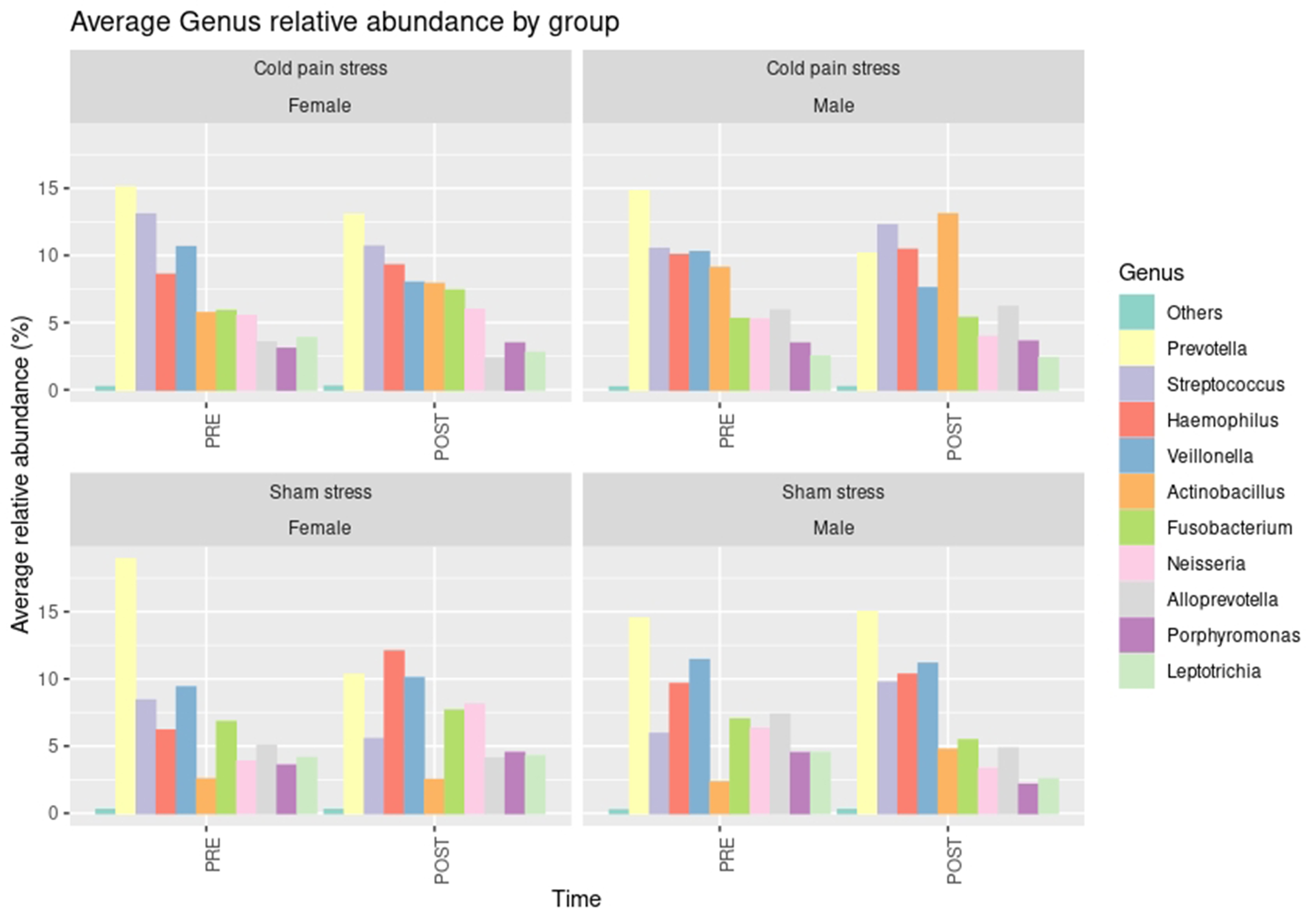

Supplement: Supplementary file 4 [file Image_2.jpeg]

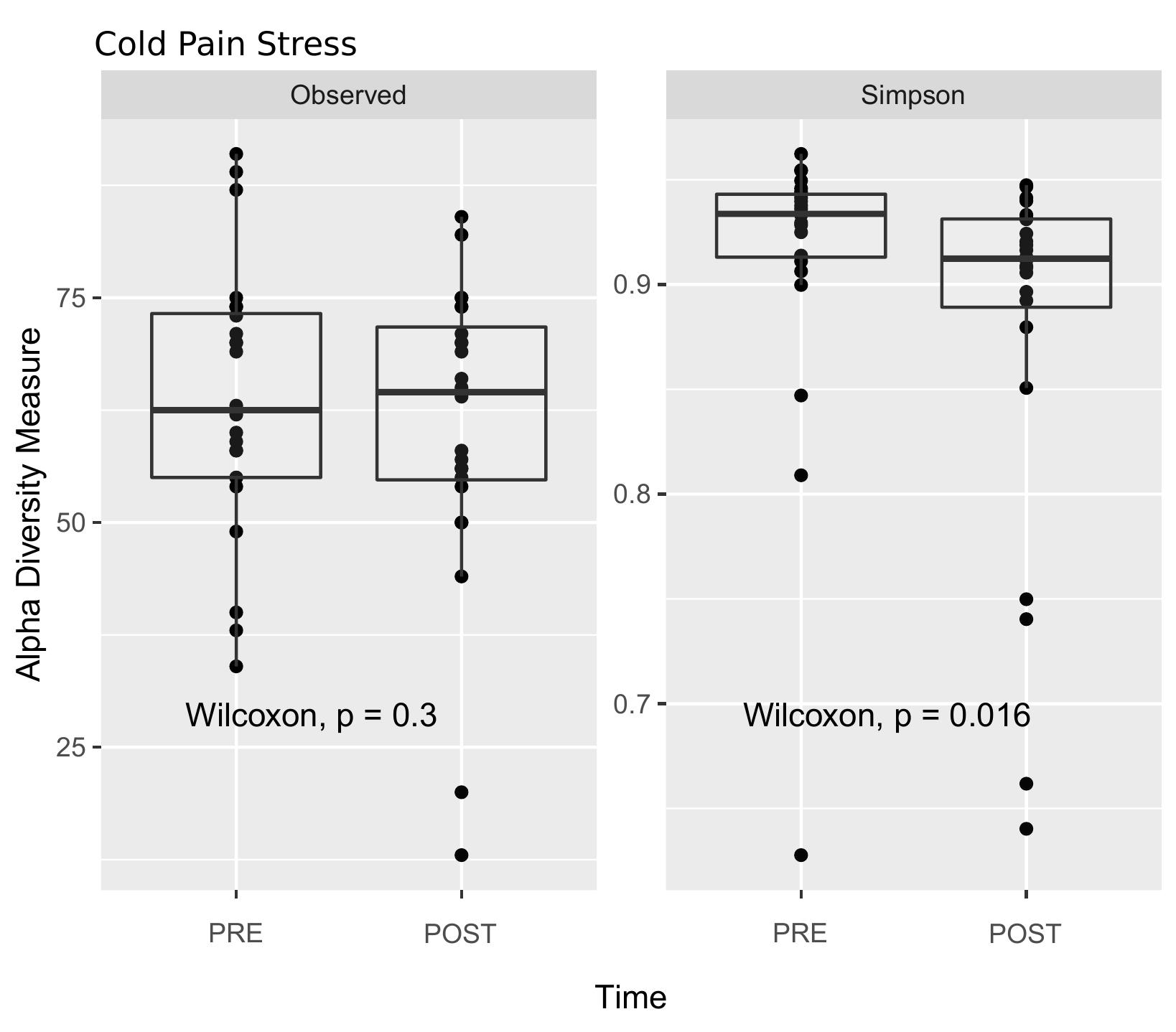

Supplement: Supplementary file 5 [file Image_3.jpeg]

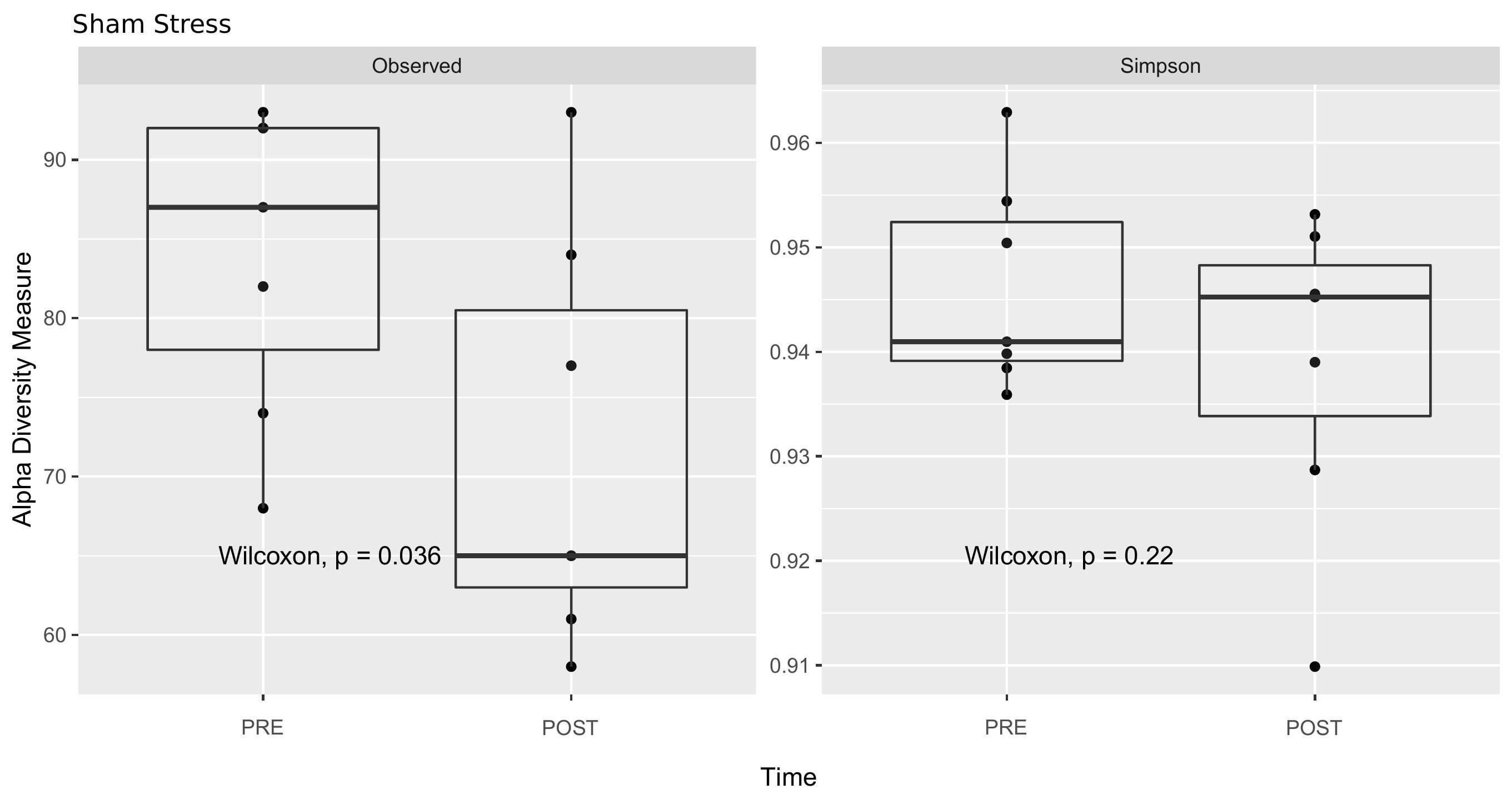

Supplement: Supplementary file 6 [file Image_4.jpeg]
